# Synergistic Potentiation of Antimicrobial and Antibiofilm Activities of Penicillin and Bacitracin by Octyl Gallate, a Food-Grade Antioxidant, in *Staphylococcus epidermidis*

**DOI:** 10.3390/antibiotics11121775

**Published:** 2022-12-08

**Authors:** Pitchaya Santativongchai, Phitsanu Tulayakul, Yinduo Ji, Byeonghwa Jeon

**Affiliations:** 1 Division of Environmental Health Sciences, School of Public Health, University of Minnesota, St. Paul, MN 55108, USA; 2 Bio-Veterinary Sciences (International Program), Faculty of Veterinary Medicine, Kasetsart University, Bangkok 10900, Thailand; 3 Department of Veterinary Public Health, Faculty of Veterinary Medicine, Kasetsart University, Kamphaeng Saen Campus, Nakhon Pathom 73140, Thailand; 4 Department of Veterinary and Biomedical Sciences, College of Veterinary Medicine, University of Minnesota, St. Paul, MN 55108, USA

**Keywords:** *Staphylococcus*, antimicrobial synergy, biofilm, antioxidant

## Abstract

*Staphylococcus epidermidis* is a major nosocomial pathogen that frequently forms biofilms on indwelling medical devices. This study aimed to investigate the synergistic antimicrobial and antibiofilm activities of octyl gallate (OG) in combination with penicillin and bacitracin against *S. epidermidis*. Antimicrobial synergy was assessed by conducting checkerboard titration assays, and antibiofilm activity was determined with biofilm assays and fluorescence microscopy analysis. The presence of 8 µg/mL of OG increased both the bacteriostatic and bactericidal activities of penicillin and bacitracin against *S. epidermidis*. It lowered the minimum inhibitory concentration (MIC) and minimum bactericidal concentration (MBC) of penicillin by eight-fold and those of bacitracin by four-fold. Moreover, when used with penicillin or bacitracin, OG significantly decreased the level of biofilm production by preventing microcolony formation. Furthermore, OG significantly permeabilized the bacterial cell wall, which may explain its antimicrobial synergy with penicillin and bacitracin. Together, these results demonstrate that OG, a food-grade antioxidant, can be potentially used as a drug potentiator to enhance the antimicrobial and antibiofilm activities of penicillin and bacitracin against *S. epidermidis*.

## 1. Introduction

*Staphylococcus epidermidis* is a coagulase-negative staphylococcal bacterium and is one of the most common causes of implant-associated infections [1,2]. *S. epidermidis* was previously considered a non-pathogenic member of the human skin microbiome but is recognized as an opportunistic pathogen frequently implicated in nosocomial infections [3]. This occurs primarily because the ubiquitousness of *S. epidermidis* on the skin results in the contamination of indwelling medical devices during surgery [3,4]. After invading the human body from contaminated implants, *S. epidermidis* produces biofilms for protection against host defenses and antibiotics and can eventually travel into the bloodstream [4]. Antimicrobial therapy to treat *S. epidermidis* infection depends on the severity of the infection. Systemic infections require parenteral antibiotic therapy, whereas localized infections can be treated with antibiotics [5]. Due to the widespread methicillin resistance in coagulase-negative staphylococci [6], the empiric therapy of choice is intravenous vancomycin [5], whereas β-lactam antibiotics are used to treat methicillin-susceptible infections [5]. However, multidrug-resistant *S. epidermidis* is becoming increasingly prevalent [7,8].

Developing antibiotic potentiators is a novel approach to the control of antibiotic resistance that utilizes existing antibiotics [9]. Drug potentiators are not necessarily antibiotics but can restore or increase the antimicrobial activity of antibiotics when delivered with antibiotics [10]. A well-known example is clavulanic acid, a β-lactamase inhibitor, which is commonly used in combination with amoxicillin to prevent the enzymatic degradation of the antibiotic by β-lactamase-producing bacteria. This strategy can revitalize antibiotics that can no longer be used due to widespread resistance [11,12]. Our previous studies demonstrated that octyl gallate (OG) enhanced the antimicrobial and antibiofilm activities of bacitracin, a polypeptide antibiotic widely used in topical antibiotic ointments to treat skin infections [13,14], against methicillin-resistant *Staphylococcus aureus* (MRSA) [15,16]. Moreover, OG is capable of sensitizing MRSA to β-lactams [17]. OG is an antioxidant used as a food additive to prevent lipid oxidation of high-fat food [18]. Our previous results suggest that OG can be potentially used as a drug potentiator to inhibit the growth of MRSA using currently available antibiotics.

Our previous studies about MRSA gave rise to a research question about whether OG can also generate antimicrobial and antibiofilm synergy in other staphylococcal pathogens. In this study, we demonstrate that OG increases the antimicrobial and antibiofilm activities of β-lactams and bacitracin in *S. epidermidis*, an important pathogen of the staphylococcal genus.

## 2. Results

### 2.1. Antimicrobial Synergy of OG and Antibiotics against S. epidermidis

**Figure 1:**
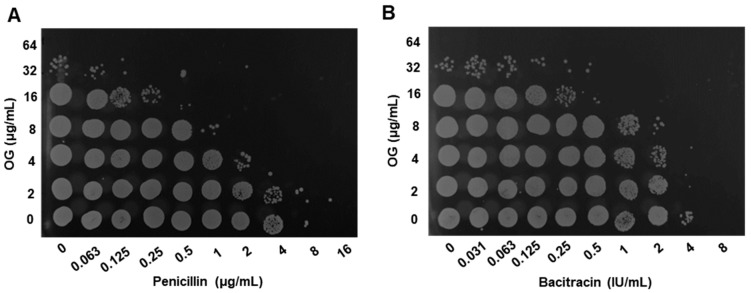
Synergistic bactericidal activity of octyl gallate (OG) in combination with penicillin (**A**) and bacitracin (**B**). White spots show bacterial growth, and empty space indicates growth inhibition.

Antimicrobial synergy was evaluated by comparing antimicrobial susceptibility in the presence and absence of OG. Notably, OG enhanced the antimicrobial activity of bacitracin and penicillin against *S. epidermidis* (Table 1). For instance, the presence of 8 µg/mL of OG reduced the minimum inhibitory concentration (MIC) and minimum bactericidal concentration (MBC) of penicillin by eight-fold and those of bacitracin by four-fold (Table 1). However, OG did not enhance the activity of ciprofloxacin, gentamicin, tetracycline, and erythromycin, which suggests that the synergy of OG is specific to penicillin and bacitracin in *S. epidermidis*. Moreover, OG and antibiotic combinations significantly reduced the viability of *S. epidermidis* (Figure 1), indicating that the synergy enhances the bactericidal activity of penicillin and bacitracin. We determined the fractional inhibitory concentration (FIC) and fractional bactericidal concentration (FBC) indices to confirm whether the enhanced antimicrobial effects are synergistic. Notably, the indices showed that combinations of OG and penicillin or bacitracin in broad concentration ranges were synergistic (i.e., ≤0.5; Table 2) [19]. Regarding the FIC index, the presence of only 2 µg/mL of OG generated antimicrobial synergy with 4 µg/mL of penicillin or 4 IU/mL of bacitracin (Table 2). These results demonstrate that OG significantly enhances the antimicrobial activities of penicillin and bacitracin in *S. epidermidis*.

### 2.2. Permeabilization of Cell Walls by OG in S. epidermidis

**Figure 2:**
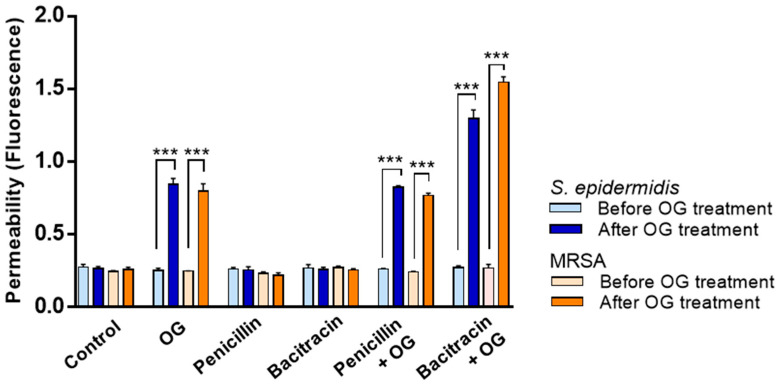
Increased cell wall permeability by octyl gallate (OG) in *Staphylococcus epidermidis* and methicillin-resistance *Staphylococcus aureus* (MRSA). The results show the means and standard deviations of fluorescence values of propidium iodide from three samples in a single experiment before and after addition of 40 µg/mL OG or PBS (control). The experiment was repeated three times and produced similar results. The concentrations of penicillin and bacitracin used in the assay were 4 µg/mL and 4 IU/mL, respectively. One-way ANOVA followed by Bonferroni’s Multiple Comparison Test was used for statistical analysis. ***: *p* < 0.01.

Our previous study shows that OG permeabilizes the bacterial cell wall in MRSA, which can increase the access of antimicrobials to the cellular targets [17]. We hypothesized that OG may increase the activity of penicillin and bacitracin against *S. epidermidis* by altering the permeability of the bacterial cell wall. Remarkably, OG significantly increased the cell wall permeability in *S. epidermidis* to a degree comparable to what was observed in MRSA (Figure 2). Although bacitracin alone did not alter the cell wall permeability, bacitracin and OG combinations significantly increased permeability (Figure 2), confirming the cell wall permeabilizing activity of OG. These results suggest that OG potentiates penicillin and bacitracin in *S. epidermidis* by permeabilizing the bacterial cell wall.

### 2.3. Antibiofilm Synergies of OG and Antibiotics against S. epidermidis

**Figure 3:**
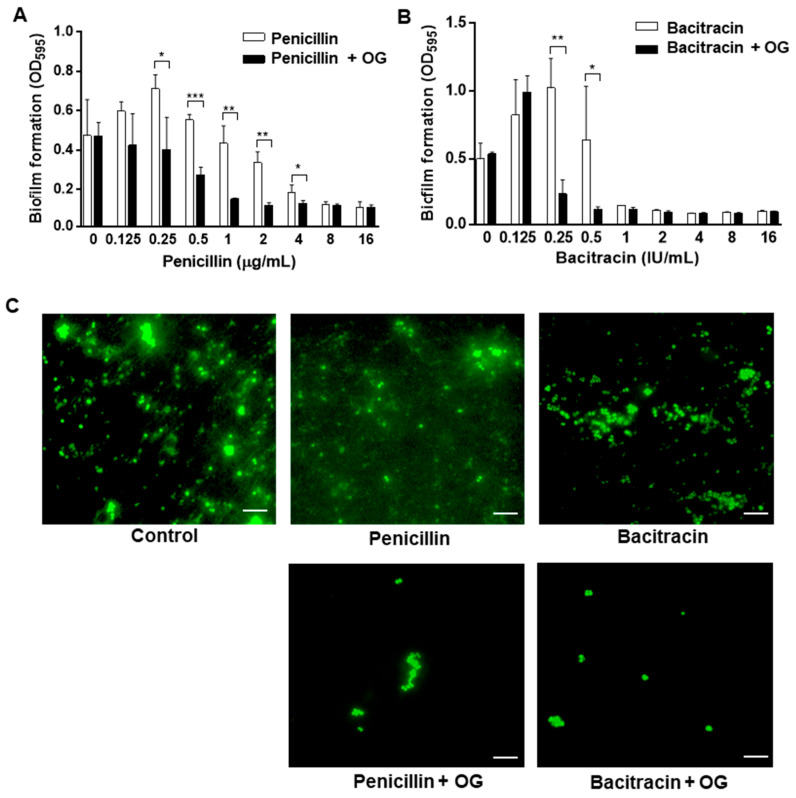
Synergistic inhibition of *Staphylococcus epidermidis* biofilms by penicillin (**A**) and bacitracin (**B**) in combination with 4 µg/mL octyl gallate (OG). The presented data are the means and standard deviations of three samples in a single experiment. The experiment was repeated three times and produced similar results. Statistical significance was determined with Student’s *t*-test by comparing samples treated with an antibiotic alone and those treated with an antibiotic and OG. *: *p* < 0.05, **: *p* < 0.01, ***: *p* < 0.001. (**C**) Fluorescence microscopy observation of *S. epidermidis* biofilms stained with SYTO 9. The scale bar indicates 5 µm.

Since OG generated significant antimicrobial synergy when combined with penicillin or bacitracin, we hypothesized that the enhanced antimicrobial activity of the OG and antibiotic combinations can inhibit biofilm production more effectively than antibiotics alone. Consistent with the hypothesis, the combinations of OG and penicillin or bacitracin synergistically increased antibiofilm activities in *S. epidermidis* (Figure 3). Antibiotics alone inhibited biofilms when used at high concentrations; however, the presence of OG allowed these antibiotics to prevent biofilm development even at low concentrations (Figure 3). Compared to non-treated controls, penicillin prevented biofilm formation when used at ≥8 µg/mL, whereas the supplementation with 4 µg/mL of OG enabled 1 µg/mL of penicillin to inhibit biofilm production (Figure 3A). Similarly, 1 IU/mL of bacitracin was needed to prevent biofilm formation, whereas 0.25 IU/mL of bacitracin could inhibit biofilm production in the presence of 4 µg/mL of OG (Figure 3B). Additionally, it is noteworthy that a certain threshold antibiotic level is needed to generate synergy. Despite the presence of OG, 0.125 IU/mL of bacitracin did not affect biofilm formation, whereas 0.5 IU/mL of bacitracin synergistically reduced biofilm production in combination with OG (Figure 3B). Moreover, fluorescence microscopy observations showed that combinations of OG with penicillin or bacitracin substantially reduced the establishment of biofilms at 48 h (Figure 3). Compared to controls, combinations of OG with penicillin or bacitracin significantly reduced the development of microcolonies, which is an initial step of biofilm formation (Figure 3). These data demonstrate that OG enhances the antibiofilm activity of penicillin and bacitracin against *S. epidermidis*.

## 3. Discussion

*S. epidermidis* is one of the most common pathogens causing infections associated with medical implants and catheters [1,2]. The findings in this study demonstrate that OG enhances the activity of penicillin and bacitracin against *S. epidermidis*, potentially providing a new adjuvant approach to the control of *S. epidermidis* using OG, an antioxidant approved by the U.S. Food & Drug Administration (FDA) for food application [18]. We previously demonstrated that even at low concentrations, OG significantly increases the susceptibility of *S. aureus* to penicillin (ca. >256-fold based on MIC) and bacitracin (ca. 2,000,000-fold based on MBC) [15,17]. Based on MIC and MBC changes, OG has relatively less antimicrobial synergy in *S. epidermidis* compared to the synergy observed in MRSA. However, our results suggest that OG can be used as an antimicrobial adjuvant to combat antibiotic-resistant staphylococcal pathogens using existing antibiotics.

The synergistic activity of OG can be ascribed to increased permeability in the bacterial cell wall in *S. epidermidis* (Figure 2), which may enable antibiotics to access their cellular targets more effectively. The mechanisms of action of β-lactams and bacitracin are commonly associated with the inhibition of the bacterial cell wall. Penicillin inhibits the synthesis of the peptidoglycan layer by modifying the active site of penicillin-binding proteins [20]. Bacitracin interrupts the synthesis of the peptidoglycan layer by inhibiting the dephosphorylation of undecaprenyl pyrophosphate [21]. The results of our permeability assay demonstrate that OG permeabilizes the bacterial cell wall in MRSA and *S. epidermidis* (Figure 2), which may partly explain why OG is synergistic in combination with the antibiotics targeting the bacterial cell wall. In *S. epidermidis*, OG was not synergistic with antibiotics other than bacitracin and penicillin, whereas OG also generates synergy with antibiotics of other classes, such as gentamicin, tetracycline, and erythromycin in MRSA [15,17]. This indicates that the mechanisms of antimicrobial synergy by OG other than the cell wall permeabilization can be different between these two pathogenic species. The elucidation of molecular mechanisms for the synergy still awaits future studies.

*S. epidermidis*, as a common member of the human skin microbiome, can be introduced to catheters and implants during surgery and can develop biofilms on the surface of these indwelling medical devices [2,4,22]. Indwelling medical devices are vulnerable to biofilm formation by pathogens, and bacterial cells in biofilms are highly tolerant to antibiotics, which makes the eradication of biofilms extremely difficult [23]. Our previous study showed that OG significantly increases the antibiofilm activity of bacitracin against MRSA [16]. Similarly, the findings in this study demonstrate that OG also enhances the antibiofilm activities of penicillin and bacitracin (Figure 3). Since biofilm formation is crucial for staphylococcal infections, synergistic antibiofilm activity is an important feature related to the potential therapeutic application of OG to the control of staphylococcal infections.

In conclusion, the results presented in this study suggest that OG, a food-grade antioxidant, can be used as a drug potentiator of penicillin and bacitracin to treat infections caused by the two major staphylococcal pathogens, MRSA and *S. epidermidis*. Bacitracin is an antimicrobial peptide widely used in topical antimicrobial ointments [13]. Since *S. epidermidis* and MRSA are commonly related to skin infection [24,25], enhanced antimicrobial and antibiofilm activities using OG as a drug potentiator can be a novel strategy to improve the treatment of skin infections. Additionally, the approach can be potentially used to prevent the establishment of biofilms on indwelling medical devices by staphylococcal pathogens using existing antibiotics.

## 4. Materials and Methods

### 4.1. Bacterial Strain and Culture

*S. epidermidis* ATCC 12228 and MRSA ATCC 33593 were purchased from the American Type Culture Collection (ATCC, Manassas, VA, USA). These strains were aerobically cultured on Brain Heart Infusion (BHI) media (Becton, Dickinson and Company, Sparks, MD, USA) at 37 °C.

### 4.2. Antimicrobial Susceptibility Tests

MIC was measured using the broth dilution method [26]. MBC was determined by spotting 5 µL of cultures on the 96-well plate, which was used to determine MIC, onto BHI agar plates. The plates were incubated at 37 °C aerobically overnight before evaluating viability. All chemicals, including OG and antibiotics, were purchased from MilliporeSigma (St. Louis, MO, USA).

### 4.3. Determination of Antimicrobial Synergy

The antimicrobial synergy between OG and antibiotics was determined using checkerboard titration assays as described previously [27]. Briefly, OG and antibiotics were two-fold serially diluted on row and column, respectively. Each well was inoculated with 100 µL of *S. epidermidis* suspension (ca. 5 × 10^5^ CFU per well). The plates were incubated aerobically at 37 °C overnight. Checkerboard assays for each combination were repeated three times. Synergistic effects were evaluated by calculating the FIC index [28]. Briefly, the FIC index was calculated as FIC = FIC_A_ + FIC_B_, where FIC_A_ is the MIC of agents A and B in combination divided by the MIC of agent A alone, and FIC_B_ is the MIC of agents A and B in combination divided by the MIC of agent B alone. FIC index ≤ 0.5 indicates synergy [19]. The FBC index was similarly determined based on the MBCs of OG and antibiotic combinations.

### 4.4. Biofilm Assay

Biofilm assays were conducted according to the previous studies [16,29]. Briefly, *S. epidermidis* biofilms were allowed to form in the presence of a range of combinations of OG and antibiotics, which were prepared by two-fold serial dilution. After overnight incubation, biofilms were washed twice with phosphate-buffered saline (PBS, pH 7.4) and stained with 1% crystal violet for 40 min. The dye was then removed, and biofilms were washed with PBS three times. The stained biofilms were eluted with the elution buffer (10% acetic acid and 30% methanol), and the optical density (OD) at 595 nm was measured with a microplate reader (Varioskan™ LUX, ThermoFisher, Waltham, MA, USA). The experiment was conducted three times.

### 4.5. Fluorescence Microscopic Analysis of Biofilms

Biofilms were observed with fluorescence microscopy. Biofilms were developed on a glass slide for 48 h at 37 °C under aerobic conditions, washed twice with PBS, and fixed with 4% paraformaldehyde (MilliporeSigma) for 30 min at room temperature. The biofilms were then washed with PBS and stained with SYTO 9 (LIVE/DEAD™ BacLight™ Bacterial Viability Kit, ThermoFisher Scientific, Waltham, MA, USA). After washing, biofilms were observed with a fluorescence microscope (Olympus BX53, Shinjuku, Tokyo, Japan).

### 4.6. Cell Wall Permeability Assay

The changes in cell wall permeability by OG were examined according to a previous study with slight modifications [17]. Briefly, overnight cultures of *S. epidermidis* and MRSA were washed with PBS and diluted to the OD at 600 nm of 0.5. MRSA was used as a positive control based on our previous study [17]. The bacterial culture was then incubated with 2.5 mg/mL propidium iodide (MilliporeSigma). After 10 min, OG or PBS (control) was added to the bacterial suspension to the final concentration of 40 µg/mL, and fluorescence was measured with a microplate reader (Varioskan™ LUX, ThermoFisher Scientific) with 535 nm excitation and 615 nm emission. The experiment was repeated three times.

### 4.7. Statistical Analysis

Statistical analysis was conducted using one-way analysis of variance (ANOVA), followed by Bonferroni’s posthoc test for multiple comparisons, or Student’s *t*-test to compare the levels of permeability and biofilm formation using GraphPad Prism 5.

## Figures and Tables

**Table 1:** Synergistic bacteriostatic and bactericidal activities of octyl gallate (OG) and antibiotic combinations against *Staphylococcus epidermidis*.

OG (µg/mL)	Penicillin		Bacitracin	
	MIC ^a^ (µg/mL)	MBC ^b^ (µg/mL)	MIC ^a^ (IU/mL)	MBC ^b^ (IU/mL)
0	4	8	4	8
2	2	8	2	4
4	1	4	2	4
8	0.5	1	1	2

^a^ MIC, minimum inhibitory concentration; ^b^ MBC, minimum bactericidal concentration.

**Table 2:** The FIC and FBC indices of combinations of octyl gallate (OG) with penicillin and bacitracin in *Staphylococcus epidermidis*.

OG (µg/mL)	FIC Index								FBC Index							
	Penicillin (µg/mL)				Bacitracin (IU/mL)				Penicillin (µg/mL)				Bacitracin (IU/mL)			
	0.5	1	2	4	0.5	1	2	4	0.5	1	2	4	0.5	1	2	4
2	0.750	0.625	0.563	0.500	1.000	0.750	0.563	0.500	1.250	1.250	1.125	1.063	1.000	1.000	1.000	0.563
4	0.500	0.375	0.313	0.250	1.000	0.750	0.563	0.500	0.750	0.750	0.625	0.563	1.000	1.000	1.000	0.563
8	0.375	0.250	0.188	0.125	0.750	0.500	0.313	0.250	0.500	0.500	0.375	0.313	1.000	1.000	1.000	0.531
16	0.266	0.141	0.078	0.016	0.508	0.258	0.070	0.008	0.313	0.313	0.188	0.125	0.313	0.313	0.313	0.094
32	0.250	0.125	0.063	0.016	0.500	0.250	0.063	0.008	0.281	0.281	0.156	0.094	0.281	0.281	0.281	0.063

Dark grey indicates synergy (FIC/FBC ≤ 0.500), and light grey shows no interaction (0.500 < FIC/FBC ≤ 4.000). The FIC and FBC cut-off values for synergy and no interaction are based on a previous report [19].
